# Prediction of Infantile Spasms Recurrence after ACTH Therapy

**DOI:** 10.15844/pedneurbriefs-29-12-4

**Published:** 2015-12

**Authors:** J. Gordon Millichap, John J. Millichap

**Affiliations:** 1 Division of Neurology, Ann & Robert H. Lurie Children's Hospital of Chicago, Chicago, IL; 2 Departments of Pediatrics and Neurology, Northwestern University Feinberg School of Medicine, Chicago, IL

**Keywords:** EEG, Infantile Spasms, ACTH, West syndrome

## Abstract

Investigators from Okayama University Hospital, Japan, studied the predictive value of serial EEG findings (every 2 to 4 weeks) in relapse of epileptic spasms after synthetic ACTH therapy in patients with West syndrome (WS).

Investigators from Okayama University Hospital, Japan, studied the predictive value of serial EEG findings (every 2 to 4 weeks) in relapse of epileptic spasms after synthetic ACTH therapy in patients with West syndrome (WS). EEGs were performed for more than 40 min, both awake and asleep. Thirty-nine WS patients (18 cryptogenic, and 31 symptomatic) received ACTH therapy for the first time and were followed for more than 3 years. The total duration of ACTH therapy ranged from 11 to 37 days (mean, 23.5 days). Sixteen (41%) showed seizure relapse and 23 patients (59.0%) had no seizure relapse. Immediately on completion of ACTH therapy, seizure outcome was associated with etiology (p=0.003). No seizure recurrence occurred in the cryptogenic group whereas 51.6% of the symptomatic group had recurrence of seizures. At one month after ACTH, only the presence of epileptic discharges (p=0.001) had a significant association with seizure outcome, regardless of etiology. All relapsed patients were in the symptomatic group. The time to relapse after completion of ACTH therapy ranged from 5 days to 25 months (mean: 6.6 months). The group with no epileptic discharges on EEG showed a significantly higher seizure-free rate than those with epileptic discharges at 1 month after ACTH (p=0.0091). Serial EEG findings after ACTH therapy for WS are significantly related and may be used to predict relapse of epileptic spasms. [1]

COMMENTARY. In both cryptogenic and symptomatic patients in this study, the group with no epileptic discharges on EEG at 2 weeks and at one month after the end of ACTH therapy showed a significantly better seizure control. The presence of epileptic discharges, especially multifocal spikes before 2 months’ post treatment, can be used to predict imminent relapse [1]. In the present study [1], the age at onset, and presumably of treatment, of spasms ranged from 2 to 15 months (mean: 6.7 months); the response in relation to age was not specifically determined, but the overall early age might predict a generally favorable response.

In addition to the EEG and etiology, factors important in the prediction of prognosis of WS not addressed in this study are the age at diagnosis and treatment, and the level of development at diagnosis. In a study of 21 patients treated with ACTH, the outcome was related significantly to the age at diagnosis and treatment: 80% of infants less than 1-year-old were benefited whereas only 22% of those over 1 year showed reduction in seizures and EEG improvement after ACTH therapy [2]. Patients of normal or borderline intelligence tended to respond to ACTH more frequently than did those with lowered mental development quotients <70 [2]. Predicting relapse of WS with EEG following treatment is important for clinical decision making and shares the same significance as screening for a pre-hypsarhythmia EEG pattern in at risk infants prior to the onset of infantile spasms [3].
